# Does endometrial scratching increase the rate of spontaneous conception in couples with unexplained infertility and a good prognosis (Hunault > 30%)? Study protocol of the SCRaTCH-OFO trial: a randomized controlled trial

**DOI:** 10.1186/s12884-018-2160-z

**Published:** 2018-12-29

**Authors:** B. N. Bui, H. L. Torrance, C. Janssen, B. Cohlen, J. P. de Bruin, J. E. den Hartog, P. J. Q. van der Linden, K. L. Deurloo, J. W. M. Maas, R. van Oppenraaij, A. Cantineau, C. B. Lambalk, H. Visser, E. Brinkhuis, J. van Disseldorp, B. C. Schoot, C. Lardenoije, M. van Wely, M. J. C. Eijkemans, F. J. M. Broekmans

**Affiliations:** 1 University Medical Centre Utrecht, Heidelberglaan 100, 3584 CX Utrecht, The Netherlands; 2 Groene Hart Hospital, Gouda, The Netherlands; 3 Isala Fertility Clinic, Zwolle, The Netherlands; 4 Jeroen Bosch Hospital, ’s-Hertogenbosch, The Netherlands; 5 Maastricht University Medical Centre+, Maastricht, The Netherlands; 6 Deventer Hospital, Deventer, The Netherlands; 7 Diakonessenhuis, Utrecht, The Netherlands; 8 Máxima Medical Centre, Veldhoven, The Netherlands; 9 Maasstad Hospital, Rotterdam, The Netherlands; 10 University Medical Centre Groningen, Groningen, The Netherlands; 11 Vrije Universiteit Medical Centre, Amsterdam, The Netherlands; 12 Tergooi Hospital, Hilversum, The Netherlands; 13 Meander Medical Centre, Amersfoort, The Netherlands; 14 St. Antonius Hospital, Nieuwegein, The Netherlands; 15 Catharina Hospital, Eindhoven, The Netherlands; 16 Laurentius Hospital, Roermond, The Netherlands; 17 Dutch Consortium for Healthcare Evaluation and Research in Obstetrics and Gynecology – NVOG Consortium 2.0, Amsterdam, The Netherlands

**Keywords:** Endometrial injury, Endometrial scratching, Endometrial scratch, Unexplained infertility, Endometrial receptivity, Implantation failure, Hunault > 30%, Expectant management

## Abstract

**Background:**

In the Netherlands, couples with unexplained infertility and a good prognosis to conceive spontaneously (i.e. Hunault > 30%) are advised to perform timed intercourse for at least another 6 months. If couples fail to conceive within this period, they will usually start assisted reproductive technology (ART). However, treatment of unexplained infertility by ART is empirical and can involve significant burdens. Intentional endometrial injury, also called ‘endometrial scratching’, has been proposed to positively affect the chance of embryo implantation in patients undergoing in vitro fertilization (IVF). It might also be beneficial for couples with unexplained infertility as defective endometrial receptivity may play a role in these women. The primary aim of this study is to determine whether endometrial scratching increases live birth rates in women with unexplained infertility.

**Method:**

A multicentre randomized controlled trial will be conducted in Dutch academic and non-academic hospitals starting from November 2017. A total of 792 women with unexplained infertility and a good prognosis for spontaneous conception < 12 months (Hunault > 30%) will be included, of whom half will undergo endometrial scratching in the luteal phase of the natural cycle. The women in the control group will not undergo endometrial scratching. According to Dutch guidelines, both groups will subsequently perform timed intercourse for at least 6 months. The primary endpoint is cumulative live birth rate. Secondary endpoints are clinical and ongoing pregnancy rate; miscarriage rate; biochemical pregnancy loss; multiple pregnancy rate; time to pregnancy; progression to intrauterine insemination (IUI) or IVF; pregnancy complications; complications of endometrial scratching; costs and endometrial tissue parameters associated with reproductive success or failure. The follow-up duration is 12 months.

**Discussion:**

Several small studies show a possible beneficial effect of endometrial scratching in women with unexplained infertility trying to conceive naturally or through IUI. However, the quality of this evidence is very low, making it unclear whether these women will truly benefit from this procedure. The SCRaTCH-OFO trial aims to investigate the effect of endometrial scratching on live birth rate in women with unexplained infertility and a good prognosis for spontaneous conception < 12 months.

**Trial registration:**

NTR6687, registered August 31st, 2017.

**Protocol version:**

Version 2.6, November 14th, 2018.

## Background

Unexplained infertility is diagnosed in up to 50% of infertile couples when standard fertility investigations have failed to detect any gross abnormality [1–3]. Uncertainty about the underlying reasons of the infertility often leads to use of empirical treatment that can involve significant physical, psychological and financial burdens [4]. Hence prediction models have been developed to distinguish subfertile couples with a poor prognosis for spontaneous pregnancy, for whom fertility treatment is thought to increase their chance of conception, from subfertile couples who still have a good prognosis to conceive spontaneously [5]. In the Netherlands, the prediction model developed by Hunault et al. (2004) is used to predict the chance of spontaneous pregnancy within 1 year. As stated in Dutch guidelines [3], couples with unexplained infertility and a prognosis according to Hunault of > 30%, i.e. a good prognosis, are advised to perform timed intercourse for at least another 6 months. If couples fail to conceive within 6 months to a year, they will usually start assisted reproductive technology (ART), i.e. intrauterine insemination (IUI) or in vitro fertilization (IVF) [3]. IUI or IVF treatment for this diagnosis, however, is empirical and is mainly done due to the perceived need to intervene to address unexplained infertility.

In previous research it has been postulated that defective endometrial receptivity, with altered expression of adhesive molecules and immunological factors, could be a contributing factor in the aetiology of unexplained infertility [4, 6–9]. At present, moderate-quality evidence indicates that endometrial injury (‘scratching’) is associated with an improvement in live birth rates (LBR) in women undergoing IVF with repeated failed embryo transfers [10]. Several hypotheses supporting the positive effect of scratching on pregnancy rates have been proposed, but the exact mechanism remains unclear [11]. The endometrial injury is thought to incite changes, like induction of decidualization and immunologic responses, which result in improved endometrial receptivity [10]. Since defective endometrial receptivity may be a cause of subfertility in couples diagnosed with unexplained infertility, scratching may be an effective therapy for these couples. Although the majority of studies showed a positive effect of scratching on pregnancy outcomes in women undergoing IVF, some recent studies did not find endometrial scratching to be effective in improving pregnancy outcomes in IVF [12, 13]. As significant clinical heterogeneity in study populations exist between studies, the applicability of the evidence may vary in different subgroups of subfertile women [10].

The effectiveness of scratching in couples trying to conceive through sexual intercourse or IUI was recently summarized in a review [14]. The main conclusion was that scratching may have a beneficial effect on the chance of conceiving, but that published studies have important limitations. Therefore these results should be interpreted with caution. Since publication of this review, one other study on endometrial injury in women with unexplained infertility has been published, reporting a non-significant difference in clinical pregnancy rates (CPR) between the intervention and control group (16.7 vs. 11.7%) [15]. Notably, this study was not adequately powered to detect the observed difference; a difference which may be clinically relevant. Furthermore, none of the published studies reported live birth rate as an outcome parameter, which is the most important outcome for a couple trying to conceive. Finally, there remains a paucity of evidence on the duration of the effect of endometrial scratching, making it unclear for how long the beneficial effect of the scratch is sustained. These observations underline the need for more robust studies on this topic.

The primary aim of the SCRaTCH-OFO study is to determine whether endometrial scratching in the natural cycle in women with unexplained infertility increases the chance of live birth. Parallel to this, it aims to evaluate if endometrial scratching leads to a decreased use of fertility treatment and costs needed to achieve a live birth. Finally, the RCT contains a nested cohort study in which endometrial samples will be banked to determine characteristics associated with reproductive success or failure within 12 months of follow-up.

## Method

### Study objective & design

The aim of this study is to evaluate whether endometrial scratching increases the chance of live birth in women with unexplained infertility and a good prognosis to conceive spontaneously (Hunault > 30%). Furthermore, the study seeks to determine endometrial characteristics correlating with (un) successful implantation and to develop endometrial organoids.

The SCRaTCH-OFO study is a multicentre randomized controlled trial (RCT) coordinated by the University Medical Centre Utrecht (UMC Utrecht, The Netherlands), which will be performed within the Dutch Consortium for Healthcare Evaluation and Research in Obstetrics and Gynecology. A prospective cohort study is embedded within the multicentre RCT, in which the endometrial biopsy will be banked for determination of characteristics associated with reproductive success or failure and for development of endometrial organoids that will open possibilities for further research into endometrium related disorders. Some of the study methods of this trial will be comparable to those previously described in the study protocol of our other scratching trial, in which scratching is performed in women undergoing IVF/intracytoplasmic sperm injection (ICSI) cycles [16].

### Study setting

#### Coordinating hospital, trial coordinator

University Medical Centre Utrecht, Utrecht, prof. dr. F.J.M. Broekmans.

#### Participating hospitals, local trial coordinators

Catharina Hospital, Eindhoven, prof. dr. B.C. Schoot.

Deventer Hospital, Deventer, dr. P.J.Q. van der Linden.

Diakonessenhuis, Utrecht, dr. K.L. Deurloo.

Groene Hart Hospital, Gouda, dr. C. Janssen.

Isala Fertility Clinic, Zwolle, dr. B.J. Cohlen.

Jeroen Bosch Hospital, ‘s Hertogenbosch, dr. J.P. de Bruin.

Laurentius Hospital, Roermond, drs. C. Lardenoije.

Maasstad Hospital, Rotterdam, dr. R. van Oppenraaij.

Maastricht University Medical Centre +, Maastricht, dr. J.E. den Hartog.

Máxima Medical Centre, Veldhoven, dr. J.W.M. Maas.

Meander Medical Centre, Amersfoort, drs. E.A. Brinkhuis.

St. Antonius Hospital, Nieuwegein, dr. J. van Disseldorp.

Tergooi Hospital, Hilversum, drs. H. Visser.

University Medical Centre Groningen, Groningen, dr. A. Cantineau.

Vrije Universiteit Medical Centre, Amsterdam, prof. dr. C.B. Lambalk.

#### Study population

Women from couples diagnosed with unexplained infertility and a Hunault of > 30%, who have been advised to continue timed intercourse during at least 6 months, are eligible. Inclusion criteria are as follows: female age between 18 and 38 years; primary or secondary infertility lasting at least 12 months; a regular menstrual cycle (defined as a mean cycle length of 21–35 days); at least one patent tube (diagnosed by negative Chlamydia antibody titre (CAT) and absence of risk factors for tubal disease, and/or diagnosed by hysterosalpingography or diagnostic laparoscopy); total motile sperm count > 3 million and a normal transvaginal ultrasound, which is defined as the absence of visible intracavitary pathology (e.g. polyps or intramural myomas with distortion of the uterine cavity). Exclusion criteria are: a history of lower abdominal or pelvic infection, a higher chance of intra-abdominal infection due to intestinal surgery, endometriosis grade 3 and 4, previous caesarean section with niche development, recurrent miscarriage (defined as ≥2 pregnancy losses prior to 20 weeks of gestation), the presence of untreated unilateral or bilateral hydrosalpinx, previous endometrial scratching, meno-metrorrhagia, and untreated endocrine disorders.

### Study endpoints

The primary endpoint is cumulative live birth rate, of which the status of ‘ongoing pregnancy’ should be achieved within 12 months after randomization, and is defined as the delivery of at least one live fetus ≥24 weeks of gestation. Secondary endpoints include 1) ongoing pregnancy rate, defined as the detection of a fetal heartbeat on ultrasound at a gestational age of 10–12 weeks; 2) clinical pregnancy rate, defined as a gestational sac visualized on ultrasound; 3) miscarriage rate, defined as a demise of an intrauterine pregnancy confirmed by ultrasound or histology; 4) biochemical pregnancy loss, defined as a spontaneous pregnancy demise after serum or urinary bèta-hCG had been detected, without an ultrasound evaluation; 5) multiple pregnancy rate; 6) time to pregnancy, defined as the time from randomization till a positive pregnancy test; however, only pregnancies that will reach the ‘ongoing’ status will be included in the analysis; 7) progression to IUI or IVF treatment; 8) pregnancy complications; 9) complications of endometrial scratching (e.g. abnormal bleeding, abnormal vaginal discharge, abdominal pain, fever); 10) costs; 11) endometrial tissue parameters associated with reproductive success or failure, such as endometrial gene expression profiles (EGPs).

### Sample size calculation

A total of 792 women will be included in the RCT of which we expect 150 to be included in the embedded study. Based on previous studies, the estimated difference in live birth rate is at least 10% between the patients with and without endometrial scratch (live birth rate respectively 45% vs. 35%).To detect such a difference with 80% power, 396 patients are needed per study arm, resulting in 792 patients in total. This number takes into account an estimated dropout rate of 5%.

### Recruitment, consent and randomization

Eligible women will be given oral and written information about the study by their gynaecologist or fertility physician. Subsequently, these women will receive additional counselling by the investigator or research nurse after 1 week to allow the women to make an informed decision on trial participation. To participate in the embedded study, patients will give broad consent to the use of their endometrial sample and data (i.e. for storing tissue in the Biobank to determine EGPs and to develop endometrial organoids). After obtaining written informed consent, the patient will be randomly allocated to either the scratch procedure or no scratch procedure. Randomization is performed by a third party with a web-based randomization program using random blocks with block sizes of 2, 4 and 6. Due to the nature of the intervention, patients and physicians are not blinded and a sham intervention will not be performed. A survey among women participating in a RCT comparing endometrial scratching to a placebo procedure, showed that participants allocated to the scratching arm were not adequately blinded due to the discomfort they experienced during the procedure, whereas the sham intervention group was in fact less likely to guess their trial allocation (unpublished thesis by Lensen). However, the guess of trial allocation was not associated with the probability of committing protocol violations or the pattern and timing of intercourse (unpublished thesis by Lensen). Furthermore, a sham procedure could expose the control group to some degree of endometrial scratching, thereby possibly interfering with the study outcome.

### Study procedure

In the intervention group, an endometrial scratch will be performed once before starting 6 months of timed intercourse, during the luteal phase of the natural cycle. Women are instructed to use urinary ovulation prediction tests that detect the putative luteinizing hormone (LH) surge (Ovulady, Clindia Benelux B.V., The Netherlands). The scratch will be performed 6 or 7 days after a positive urine ovulation test by a dedicated gynaecologist or fertility physician. If scheduling on this day is impossible then the scratch can also be planned 5 or 8 days after a positive ovulation test. Women will receive instructions that they need to make sure that they are not pregnant in the scratch cycle (no sexual intercourse or sexual intercourse with appropriate contraceptive methods (e.g.condoms)). After cleansing of the cervix with betadine, an endometrial biopsy catheter will be introduced into the cervix up to the uterine fundus. In most cases this will be a Pipelle de Cornier® biopsy catheter, but the type of biopsy catheter may vary per participating centre. The piston will be drawn back to the end of the biopsy catheter. Then the catheter is slowly retracted while rotating over several ranges of 360 degrees during 1 to 2 min. The protocol differs slightly for the subgroup of whom endometrial tissue will be stored. Firstly, a sterile gown, gloves and cap will be worn during the procedure in order to minimalize RNase contamination. Secondly, cleaning of the cervix will not be performed with betadine but with sterile saline solution (sodium chloride 0.9%) because the outcomes of RNA analysis could be affected by betadine. In order to minimalize RNase contamination, sterile gloves are changed for new gloves directly after the procedure. The endometrial tissue will be divided into three equal parts and each part will be stored in a Tissue Sampling Storage Tube (3 ml, Cat. No. 68–4000-00, Fluid X). In the coordinating centre (UMC Utrecht) and in one of the participating centres (Diakonessenhuis), two tubes will be snap-frozen in liquid nitrogen and one tube will be used to collect tissue for slow freezing of cells for organoid development. In the other tissue-storing centres, three tubes will be snap-frozen. The process of snap-freezing is performed within maximally 3 min from taking the biopsy. Subsequently, the snap-frozen tissue will be stored in freezers at − 80 °C until later determination of RNA profiles. Due to rapid developments in the field of genetics, the suited gene sets and techniques will be determined at the time of tissue analysis. The endometrial tissue intended for development of endometrial organoids will first be slow-frozen in medium containing dimethylsulfoxide (DMSO) and then stored in liquid nitrogen at − 196 °C until later formation of endometrial organoids. For this latter procedure the technique published by Boretto et al. (2017) [17] will be used.

**Fig. 1:**
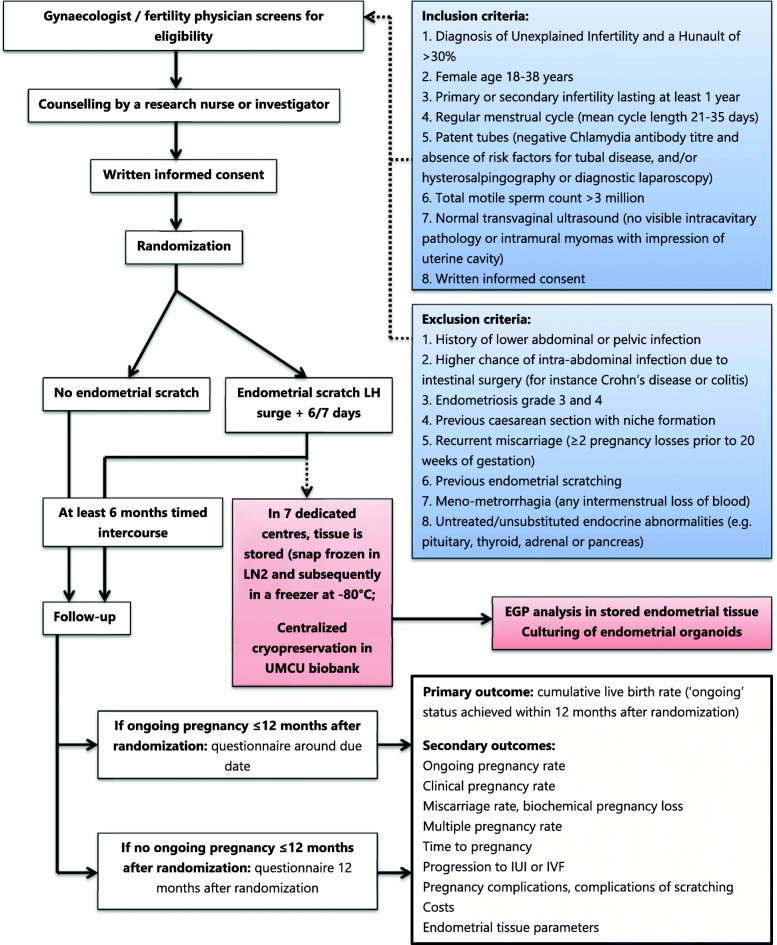
Study flowchart. LH, luteinizing hormone. LN2, liquid nitrogen. UMCU, University Medical Centre Utrecht. IUI, intrauterine insemination. IVF, in vitro fertilization. EGP, endometrial genetic profile. The boxes in pink apply to the nested cohort study

**Fig. 2:**
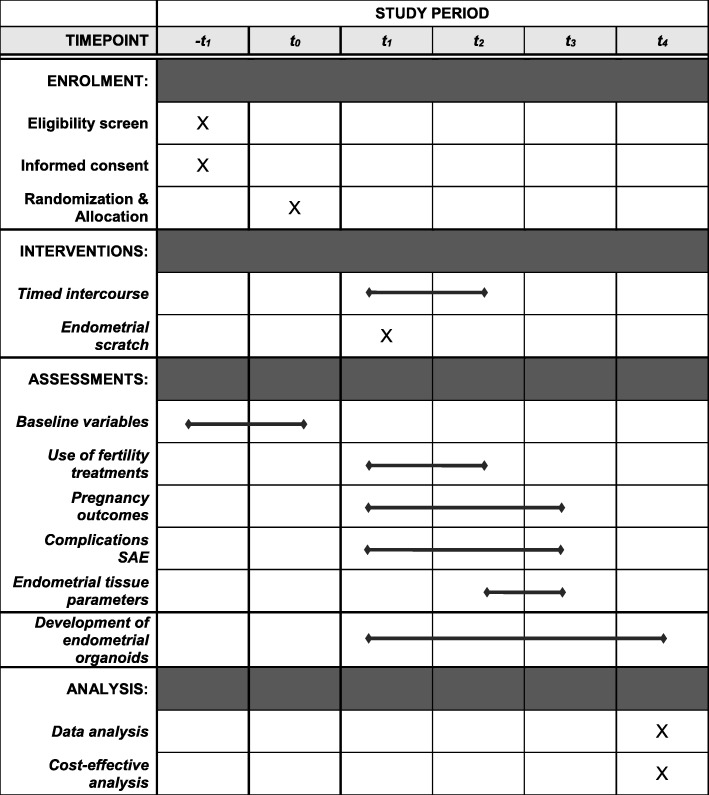
Study time schedule according to SPIRIT (Standard Protocol Items: Recommendations for Interventional Trials)-t1, pre-study period. t0, allocation. t1, post-allocation period; women allocated to the endometrial scratch will undergo this procedure in the same or next month after randomization, in the luteal phase of the natural cycle (6 to 7 days after a positive ovulation test). t2, 12 months after allocation, at which a follow-up questionnaire will be sent to all women who have completed 12 months of follow-up. t3, period in which follow-up of women with an ongoing pregnancy, within 12 months after randomization, will continue until live birth. t4, close-out period. SAE, severe adverse events

Both the intervention and control group will undergo at least 6 months of timed intercourse from the moment of randomization. An overview of the study design and a study time schedule can be found in respectively Fig. 1 and Fig. 2.

### Follow-up

The follow-up duration is 12 months from the moment of randomization. Each centre registers whether a patient has conceived during the follow-up period. When a woman has become pregnant, she will undergo ultrasounds at 7–8 weeks and 10–12 weeks of gestation, after which she will continue her prenatal visits with her midwife or gynaecologist. The outcome of her pregnancy will be obtained after her due date by a structured questionnaire.

Data on all pregnancies and further fertility treatment(s) within 12 months after randomization will be obtained by a structured questionnaire. If available, this information will also be extracted from the electronic patient file.

### Monitoring

Monitoring will be performed by a monitor from the Dutch Consortium for Healthcare Evaluation in Obstetrics and Gynaecology. Furthermore, we have installed a Data and Safety Monitoring Board (DSMB) from the Dutch Consortium for Healthcare Evaluation and Research in Obstetrics and Gynecology. Roles of the DSMB include monitoring for evidence of treatment harm, such as serious adverse events, and advising on continuation or termination of the trial. Interim analyses have not been planned.

### Data collection and data analysis

Data will be collected in a web based registration system. Database cleaning will consist of internal consistency checks and identification of database entries outside expected ranges. Analysis will primarily be conducted according to the intention-to-treat principle. In case many women allocated to the intervention group ended up not having the scratch procedure (or vice versa), a per-protocol analysis may also be performed to clarify whether there may have been an underestimation of the magnitude of effect. The researchers performing the data collection and data analysis are not blinded to group allocation.

SPSS Statistics (IBM Corporation, Armonk, NY, United States), R (R Foundation, New Zealand) and Excel (Microsoft, Washington, United States) will be used to perform the statistical analysis. The primary and secondary outcome variables (except the costs, of which analysis is described below) will be compared between the treatment arms and expressed as relative risk with 95% confidence interval. A *p*-value less than 0.05 will be considered to indicate a statistically significant difference.

### Cost-effectiveness analysis

A cost-effectiveness analysis covering a period of 12 months will be performed parallel to the clinical trial for economic evaluation, which is designed from both a healthcare and societal perspective. This cost-effectiveness analysis will be based on live birth rate and average costs per patient, taking into account any additional fertility treatments patients undergo during these 12 months. The impact on patient’s life will be expressed in quality-adjusted life years (QALY). A decision model will be used to evaluate the optimal strategy, taking into account the time to pregnancy, (in) direct costs and estimated QALY. Additionally, the incremental cost-effectiveness ratio (ICER) and long-term costs, such as delivery and perinatal costs, will be determined. Sensitivity analysis will define robustness of the results.

## Discussion

Since a study in 2003 [18] reported improved implantation rates after endometrial scratching, this procedure has been getting increasing global attention. Even though conclusive scientific evidence on its beneficial effects is still lacking, endometrial scratching is frequently being performed in women with subfertility [16]. While this intervention might be favourable for patients undergoing IVF, the effectiveness remains unclear for women with unexplained infertility trying to conceive naturally or through IUI. Studies to date show a possible beneficial effect of endometrial scratching in the latter group of women [14]. However, the quality of this evidence is very low and therefore these results should be interpreted with caution. To give clarity on this topic, robust studies are needed.

If these studies can confirm a favourable effect of endometrial scratching in increasing the live birth rate, this intervention could be offered as a simple and cost-effective treatment for unexplained infertility. Consequently, this potentially avoids some patients undergoing more expensive and invasive treatments such as IVF [19]. On the contrary, if further studies show that there is no beneficial effect of endometrial scratching on live birth rate in women with unexplained infertility, this group should not undergo an intervention with additional costs and potential risks as pain and infection.

The current study will evaluate the effect of endometrial scratching on live birth in women with unexplained infertility who are trying to conceive spontaneously. In addition, this study seeks to identify whether endometrial genetic profiles (EGPs) can predict the chance of reproductive failure within one year in couples with unexplained subfertility, as Koot et al. (2016) have shown that an endometrial 303-gene expression profile can predict repeated implantation failure (RIF) in women undergoing IVF/ICSI (PPV 100%) [20]. Defective endometrial receptivity may contribute to the aetiology of unexplained infertility. Therefore identifying an EGP, that is related to impaired endometrial receptivity, could help clinicians in counselling and guiding treatment of women with unexplained infertility. Due to rapid developments in the field of genetics, we have not determined the exact design for analysis of these EGPs yet. As the strategy might implement some new techniques or new genetic markers, it will be finalized later. Until then, tissue will be stored for future research. Lastly, development of endometrial organoids will be part of the embedded study. In the past decade there has been a rediscovery of organoids, which are three-dimensional (3D), self-organizing and genetically stable cell cultures with cell types that resemble the tissue of origin [21–23]. One of their most valuable features is that organoids recapitulate key structural and functional properties of the specific organ they have been derived from [22]. Consequently, patient-derived organoids offer possibilities to mimic pathologies in a dish and develop personalized treatments [22]. Organoids can be generated from either 1) pluripotent stem cells, like embryonic stem cells (ESC) and induced pluripotent stem cells (iPSC) or 2) organ-restricted adult stem cells (aSC) [21]. For the development of endometrial organoids, aSC are used [17, 23]. As the human endometrium is highly regenerative, these aSC can be identified in adult endometrial tissue during the process of tissue damage repair and self-renewal [21, 24]. When conditions that mimic the stem cell niche environment are created around the aSC, these stem cells are induced to form organoids [21, 25]. In the current embedded study, endometrial tissue of women with unexplained infertility will be developed into endometrial organoids after broad informed consent. In Reproductive Medicine these would be the first endometrial organoids generated from cryopreserved biopsy catheter derived endometrial tissue. Previous endometrial organoids were created from fresh, non-frozen biopsy catheter derived tissue or laparoscopically obtained biopsies [17, 23]. The development of endometrial organoids will open possibilities for further research into endometrial receptivity and other endometrial disorders.

## Abbreviations

ART: Assisted reproductive technology
CAT: Chlamydia antibody titre
CPR: Clinical pregnancy rate
EGP: Endometrial genetic profile
ICER: International cost-effectiveness ratio
ICSI: Intracytoplasmic sperm injection
IUI: Intrauterine insemination
IVF: In vitro fertilization
LBR: Live birth rate
LH: Luteinizing hormone
OFO: Standard fertility work-up (in Dutch: oriënterend fertiliteits onderzoek)
QALY: Quality-adjusted life years
RCT: Randomized controlled trial
RIF: Repeated implantation failure
RNA: Ribonucleic acid
